# Secondary Hydrogeologic Regions of the Conterminous United States

**DOI:** 10.1111/gwat.12806

**Published:** 2018-08-09

**Authors:** Kenneth Belitz, Elise Watson, Tyler D. Johnson, Jennifer Sharpe

**Affiliations:** ^1^ U.S. Geological Survey 4165 Spruance Rd., San Diego CA 92101.; ^2^ U.S. Geological Survey 405 N. Goodwin Ave., Urbana IL 61801.

## Abstract

The U.S. Geological Survey (USGS) previously identified and mapped 62 Principal Aquifers (PAs) in the U.S., with 57 located in the conterminous states. Areas outside of PAs, which account for about 40% of the conterminous U.S., were collectively identified as “other rocks.” This paper, for the first time, subdivides this large area into internally‐consistent features, defined here as Secondary Hydrogeologic Regions (SHRs). SHRs are areas of other rock within which the rocks or deposits are of comparable age, lithology, geologic or physiographic setting, and relationship to the presence or absence of underling PAs or overlying glacial deposits. A total of 69 SHRs were identified. The number and size of SHRs identified in this paper are comparable to the number and size of PAs previously identified by the USGS. From a two‐dimensional perspective, SHRs are complementary to PAs, mapped only where the PAs were not identified on the USGS PA map and not mapped where the PAs were identified. SHRs generally consist of low permeability rocks or deposits, but can include locally productive aquifers. The two maps, taken together, provide a comprehensive, national‐scale hydrogeologic framework for assessing and understanding groundwater systems.

## Abstract 2

*Article impact statement*: Identification of Secondary Hydrogeologic Regions in areas outside of Principal Aquifers expands the framework for U.S. national assessments.

## Introduction

Regional‐scale classification of groundwater systems provides a basis for understanding and management of groundwater resources (Barthel 2014; Dennehy et al. 2015). In the United States (U.S.), two classification systems have been widely used: an approach developed by Heath (1982, 1984, 1988), and subsequent work by the U.S. Geological Survey (USGS) presented in “The Ground Water Atlas of the United States” (Miller 2000). The Ground Water Atlas included a map of Principal Aquifers, which was subsequently updated by the USGS (2003). In the five paragraphs that follow, the work of Heath and the USGS are reviewed. Those studies provide the foundation for the work presented in this paper. The review of previous work is followed by two paragraphs describing the purpose and potential value of the current work.

Heath (1982 and 1984) provided a classification system for identifying groundwater regions, which were defined as areas in which the composition, arrangement, and structure of rock units are similar. Heath, building upon the work of Meinzer (1923) and Thomas (1952), identified 15 groundwater regions in the U.S., Puerto Rico, and the Virgin Islands. Heath (1988) subsequently expanded his classification system to North America, identifying 28 hydrogeologic regions. Heath's classification system has been widely used. For example, it provided the framework for describing the hydrogeology of North America (Back et al. 1988) and provided a foundation for the initial development of the widely cited DRASTIC method for assessing vulnerability to pollution (Aller et al. 1987). Heath's groundwater regions are large and focus on the general characteristics of groundwater systems that provide water supply. Heath did not explicitly identify specific aquifers or aquifer systems within those regions.

**Figure 1:**
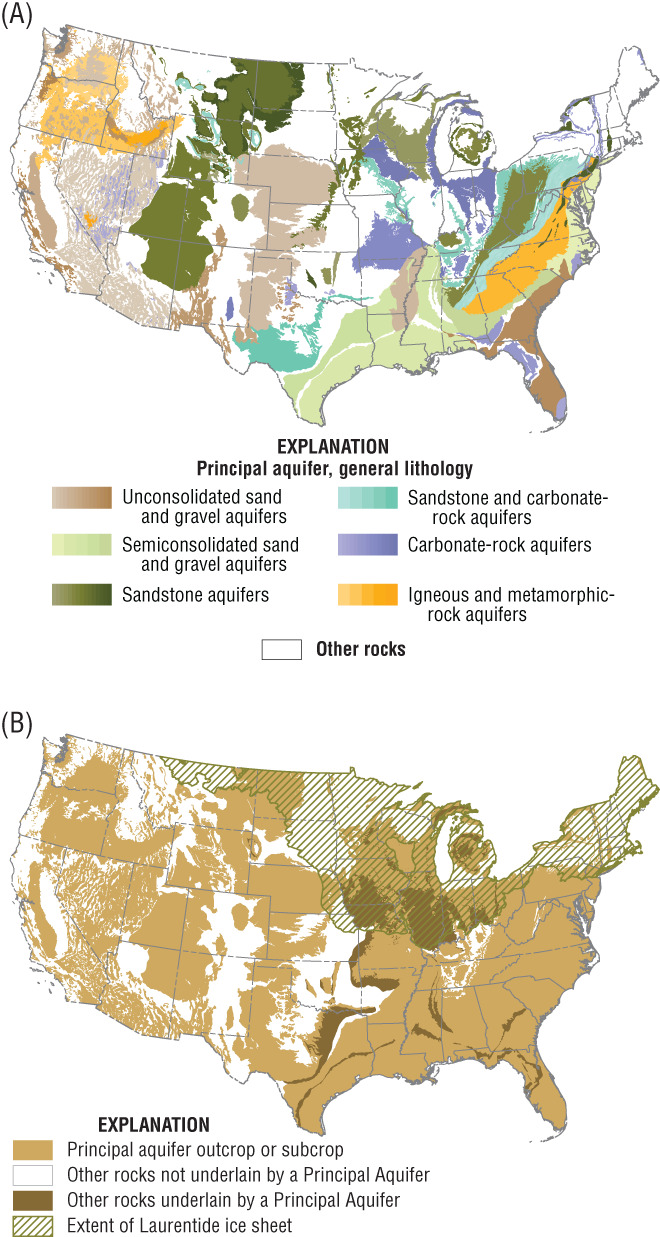
Maps of the conterminous U.S. showing: (A) Principal Aquifers, categorized by lithology, and other rocks; and (B) Principal Aquifers, areas where other rocks are and are not underlain by Principal Aquifers, and areas where glacial deposits overlie either Principal Aquifers or other rocks (as indicated by extent of Laurentide ice sheet).

The USGS (Miller 2000) subsequently published “The Ground Water Atlas of the United States” which described the “location, extent, and geologic and hydrologic characteristics of the most productive aquifers in the United States.” The productive aquifers were identified as principal aquifers (PAs) and were characterized as belonging to one of six lithologic categories (Figure 1A). Areas outside of PAs were simply identified as “not a principal aquifer” by Miller (2000) and as “other” on the USGS PA map (USGS 2003). Areas mapped as other were described by the USGS (2003) as “rocks that are minimally permeable but may contain locally productive aquifers.” For the purposes of discussion, areas outside of PAs are defined here as “other rocks.”

A total of 62 PAs were identified in the 50 states, Puerto Rico, and the Virgin Islands, with 57 located in the conterminous U.S. (USGS 2003). The areas mapped as PAs (USGS 2003) only include those areas where the PAs outcrop at the surface or subcrop beneath surficial deposits. However, many of the PAs are present at depth and underlie other PAs or underlie areas mapped as other rocks. The areas where the PAs are “buried” beneath other PAs or other rocks are not shown on the USGS PA map. The USGS PA map identifies an additional aquifer system, glacial deposit aquifers, and maps those as an overprint indicating that they can overlie either PAs or other rocks. In contrast to Heath, the USGS PA map does not identify stream‐valley aquifers (Sargent et al. 2008).

The USGS PA map has been used as a framework for assessing groundwater resources and for understanding groundwater systems. For example, USGS assessments of groundwater availability (Reilly et al. 2008) and groundwater quality (Rowe et al. 2013; DeSimone et al. 2014) have been organized on the basis of PAs. Examples of process‐based research conducted in the context of PAs include studies of redox (McMahon and Chapelle 2008), vulnerability to nitrate contamination (Gurdak and Qi 2012), recharge (McMahon et al. 2011), groundwater sustainability (Scanlon et al. 2012), and groundwater level response to climate variability (Kuss and Gurdak 2014).

Heath's groundwater regions and the USGS PA map have both been used to develop and (or) evaluate classification systems that characterize surface‐groundwater interaction. Wolock et al. (2004), building upon concepts developed by Winter (2001), identified 20 Hydrologic Landscape Regions (HLRs) in the U.S. Bedrock permeability was one of the variables considered in the statistical analysis (Wolock et al. 2004). Seven permeability classes, based on the USGS PA map, were recognized: six corresponding to the six lithologic categories and a seventh to other rocks. Glacial deposits were not included in the analysis. Santhi et al. (2008) subsequently evaluated the relationship between base flow indices and the HLRs, and used Heath's (1984) groundwater regions as a framework for discussing results. Dahl et al. (2007) explicitly incorporated Heath's (1982) classification criteria into a typology for characterizing groundwater‐surface water interaction in Denmark.

The purpose of this paper is to subdivide, within the conterminous U.S., other rocks as identified on the USGS PA map (Figure 1A) into logically mapped subareas. It is important to subdivide these areas because other rocks account for about 40% of the conterminous U.S., and because about 3% of groundwater pumping in the U.S. is from other rocks (Maupin and Barber 2005). Also, stream base flow is not limited to areas mapped as PAs. Toward that end, regional‐scale SHRs are identified as the basic subdivision of areas previously identified as other rocks.

From the perspective of a two‐dimensional map, SHRs are complementary to Principal Aquifers. SHRs are only mapped in areas identified as other rocks on the USGS PA map, and they are not mapped in areas that are identified as PAs on the USGS PA map (Figure 1A). SHRs can include locally productive aquifers or can consist entirely of low permeability rocks or deposits. With the addition of SHRs, all areas of the conterminous U.S. belong to an internally‐consistent mapped feature—either a PA or an SHR—thus providing a comprehensive, national‐scale hydrogeologic framework. The newly defined framework is at a finer spatial scale than Heath's groundwater regions. The framework of PAs and SHRs can be used to assess groundwater resources, to understand groundwater systems, and to help characterize the interaction of groundwater with surface water.

## Methodology

**Figure 2:**
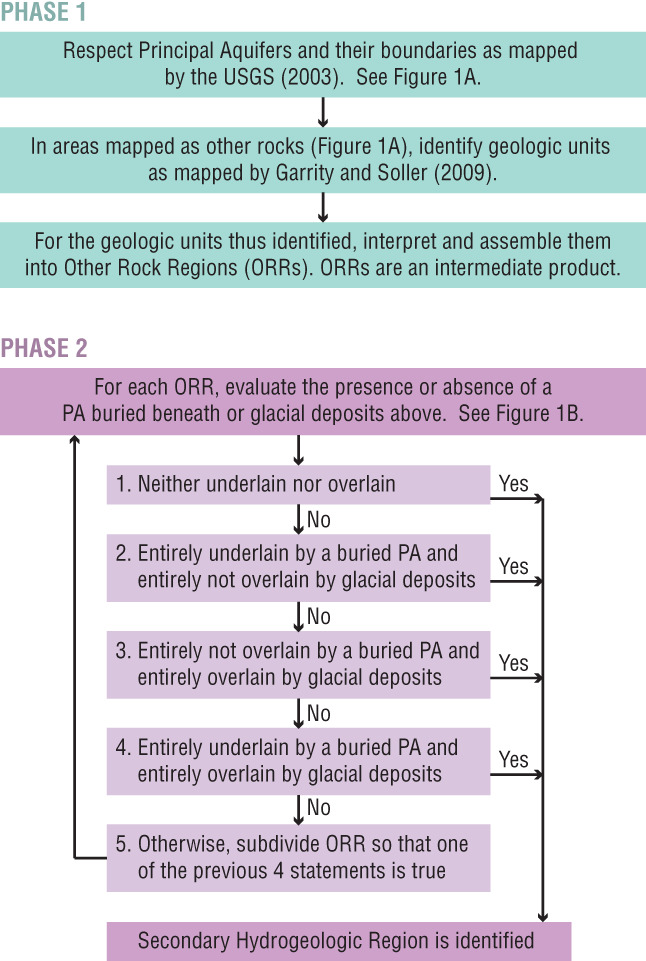
Flow charts showing the sequence of steps for identification of Other Rock Regions (Phase 1) and Secondary Hydrogeologic Regions (Phase 2).

SHRs were identified in two phases (Figure 2). In the first phase, Other Rock Regions (ORRs) were defined as regions underlain by geologic units of comparable age, lithology, and geologic or physiographic setting. ORRs were an intermediate product. In the second phase, ORRs were evaluated relative to the presence of PAs that may underlie the ORRs and (or) glacial deposits that may overly the ORRs. The presence or absence of stream‐valley aquifers overlying an SHR was not considered, which is consistent with the identification of PAs. Identification and mapping of ORRs and SHRs was facilitated using digital databases and geographic information system tools.

In the first phase, areas identified as other rocks on the PA map (USGS 2003) were intersected with geologic units from the Geologic Map of North America (Reed et al. 2005; Garrity and Soller 2009). Polygons and segments of polygons from the Geologic Map that were located within the area of other rocks were retained; polygons and segments of polygons from the Geologic Map that were located in areas of PAs were eliminated. The intersection resulted in 310 geologic units distributed across 14,000 polygons within the conterminous U.S.; segments of polygons from the Geologic Map were taken as polygons at this stage. The area of the average polygon was about 230 km^2^. Each polygon was evaluated from a hydrogeologic perspective and compared to neighboring polygons for the purposes of defining ORRs. ORRs were not assembled through the development of an algorithm or script, but rather through inspection.

Polygons were assembled into ORRs primarily on the basis of age and lithology, which are the primary descriptors of geologic units (Garrity and Soller 2009). Divisions of geologic age were as small as periods and as large as eras, largely reflecting the precision associated with the geologic units. Three lithologic classes were recognized: sedimentary (clastic or carbonate), crystalline (plutonic or metamorphic), or volcanic (Tertiary or Quaternary). Rocks of Archean and Proterozoic age were classified as crystalline. Lithologic categorization was based primarily on the relatively coarse‐resolution Geologic Map of North America (Garrity and Soller 2009), but also on the relatively fine‐resolution State Geologic Map Compilation (Horton et al. 2017). The Geologic Map of North America was given priority because it provides consistency across state boundaries, whereas the state‐scale geologic maps do not. However, the Geologic Map of North America in some cases does not provide sufficient lithologic information, and therefore the state‐scale geologic maps needed to be used. For example, the Geologic Map of North America does not distinguish between sedimentary and metasedimentary rocks. In some situations, geologic units of disparate age and lithology were assigned to ORRs that would otherwise consist of geologic units of similar age and lithology; in those cases, the disparate geologic units were relatively small and contiguous with the larger ORR.

Assemblage of polygons into ORRs also depended on the location of the polygons within geologic provinces, and other geologic or physiographic features. Four geologic provinces were recognized (Reed and Bush 2007): Cordilleran Mountain System, Central Interior, Coastal Plain, and Appalachian‐Ouachita Mountain Systems. Additional geologic features included sedimentary basins and other structures (Frezon and Finn 1988; Coleman and Cahan 2012). Physiographic regions were identified from Fenneman and Johnson (1946). The boundaries of the provinces, sedimentary basins and other structures, and physiographic regions are generalized and were used only as a guide for assembling polygons into ORRs. A total of 66 ORRs were identified, with an average size of about 48,000 km^2^.

In the second phase, the presence of PAs that might be buried beneath ORRs was taken into account (Figure 1B). The presence of PAs beneath ORRs was established by comparing the full extent of the PAs as identified in various chapters of the Groundwater Atlas (Miller 2000) to the areas mapped as outcrops on the PA map (USGS 2003). The full extents of two PAs in the Northern Great Plains—Lower Cretaceous and Paleozoic—were not utilized because those PAs contain saline water at depth (Whitehead 1996).

Also in the second phase, the presence of glacial deposits that might overlie ORRs was considered (Figure 1B). The extent of glacial deposits was taken as the southernmost extent of the Laurentide ice sheet (Arnold et al. 2008) because most of the surficial deposits within that area are mapped as glacial sediments (Soller et al. 2009; Soller and Reheis 2004). The area within the extent of the Cordilleran ice sheet was excluded because most of the surficial deposits within that area are mapped as residual materials and other non‐glacial sediments.

Each ORR was then evaluated with respect to the presence or absence of underlying PAs and (or) overlying glacial deposits (Figure 2). If the relationships were consistent across the entire ORR, then the ORR was identified as an SHR. If the relationships were not consistent, then the ORR was subdivided so that the relationships were consistent; at this stage, previously identified polygons from Phase 1 were subdivided as needed. In some cases, subdivided ORRs were combined with other subdivided ORRs to form an SHR. A total of 69 SHRs were identified with an average area of 46,000 km^2^.

Each of the SHRs was classified using three criteria: (1) presence or absence of underlying PAs or overlying glacial deposits, (2) primary lithology, and (3) geologic province. The first criterion is referred to as Type (Table 1). The classification of an SHR is based on the predominant characteristic across the SHR. Geodatabases containing information about the ORRs and SHRs are available (Belitz et al. 2018).

**Table 1:** Classification of Secondary Hydrogeologic Regions (SHRs) by Type, Where Type Indicates the Relationship Between an SHR and the Presence or Absence of Underlying Principal Aquifers (PAs) and (or) Overlying Glacial Deposits

		Overlain by Glacial?	
		No	Yes
Underlain by PA?	No	Type NN	Type NY
	Yes	Type YN	Type YY

In the discussion section, we provide some information on community supply wells. The latitude and longitude for each well was obtained from Price and Maupin (2014). These data were intersected with SHR boundaries to obtain summaries.

## Results

**Figure 3:**
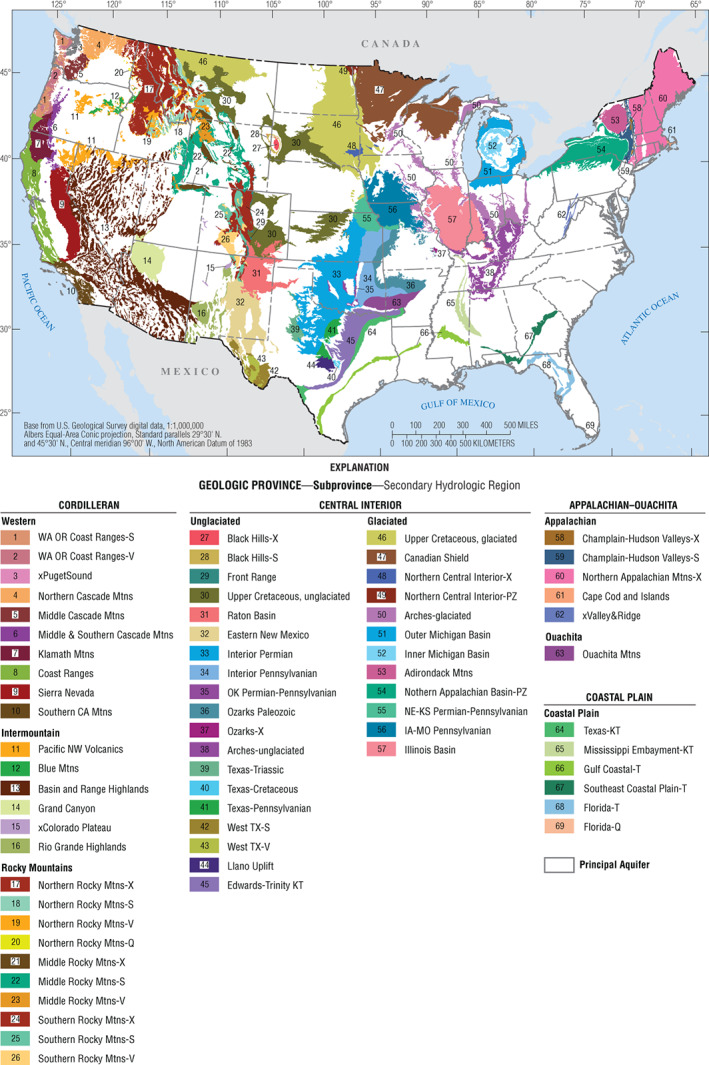
Map showing the 69 Secondary Hydrogeologic Regions of the conterminous United States. Table A1 provides information for each of the 69 SHRs.

A total of 69 SHRs were recognized within the conterminous U.S. (Figure 3), and account for all of the areas mapped as other rocks on the PA map (Figure 1). For the purposes of illustration, the Cordilleran geologic province was divided into three subprovinces: Western, Intermountain, and Rocky Mountains. The Central Interior was divided into two subprovinces: glaciated Central Interior and unglaciated Central Interior. The explanation box for Figure 3 is organized from west to east and from north to south: by geologic province, then by subprovince, and then by SHR. Table A1 provides information—province, subprovince, area, lithology, and type—for each of the 69 SHRs.

The names of the SHRs (Figure 3) were selected to reflect characteristics unique. Many of the SHRs are named for a geologic feature, such as a sedimentary basin, or physiographic region: if no lithologic or age information is included in the name, then the geologic feature or physiographic region generally consists of geologic units of comparable lithology and age; otherwise, the SHR name includes an indicator of lithology or age. Other SHRs are named for geologic units of a given age; the names of these SHRs typically include a province or geographic location followed by the age of the geologic units that comprise the SHR. Several of the SHRs are logical extensions of previously identified PAs; these SHRs have an “x” as the first letter of the SHR name followed by the name of the associated PA.

**Figure 4:**
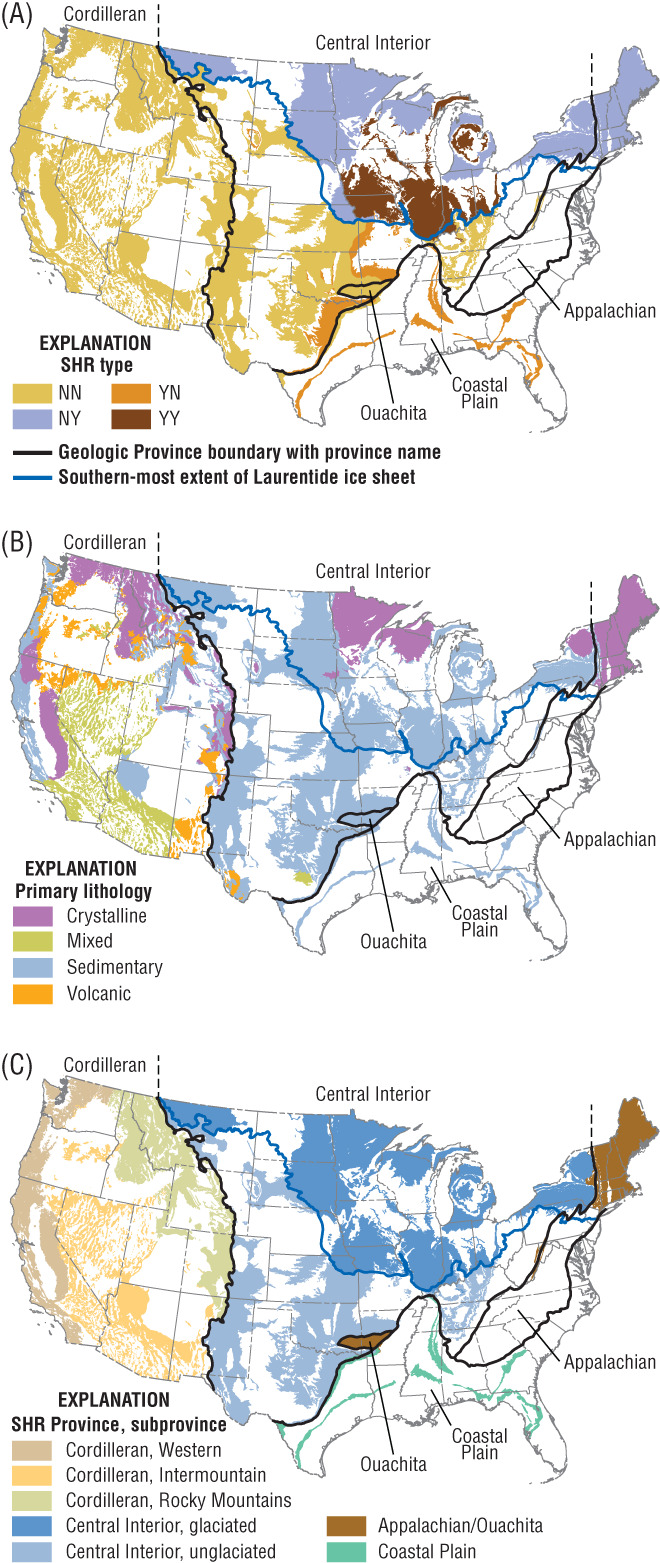
Maps showing Secondary Hydrogeologic Regions (SHRs) classified by (A) type (explanation of type provided in Table 1); (B) primary lithology; and (C) geologic province and subprovince.

Table 2 provides summary information—number of SHRs and area—based on type, lithology, and geologic province. Figure 4 shows maps of the SHRs based on the three criteria. The most common type of SHR is NN (Table 1) and the most common lithology is sedimentary. Table 3 expands on the summary provided in Table 2.

**Table 2:** Number and Size (Square Kilometers) of Secondary Hydrogeologic Regions Based on: Type, Primary Lithology, and Geologic Province

	Number	Area
Presence of PA Below or Glacial Deposits Above		
Type NN	44	1,810,000
Type NY	12	860,000
Type YN	9	200,000
Type YY	4	310,000
Primary Lithology		
Sedimentary	42	2,000,000
Crystalline	14	730,000
Volcanic	9	190,000
Mixed	4	280,000
Geologic Province		
Central Interior	31	1,840,000
Cordilleran	26	1,000,000
Appalachian‐Ouachita	6	210,000
Coastal Plain	6	100,000

Note(s): Area is rounded to the nearest 10,000 km^2^. PA, principal aquifer. Explanation of type is provided in Table 1. The total area of the conterminous U.S. is about 8 million square kilometers.

**Table 3:** Number of Secondary Hydrogeologic Regions within Each of the Geologic Provinces as a Function of Primary Lithology and Type

	Type			
	NN	NY	YN	YY
Central Interior				
Sedimentary	11	5	4	4
Crystalline	2	3	0	0
Volcanic	1	0	0	0
Mixed	1	0	0	0
Cordilleran Mountain System				
Sedimentary	9	0	0	0
Crystalline	6	0	0	0
Volcanic	8	0	0	0
Mixed	3	0	0	0
Appalachian‐Ouachita Mountain System				
Sedimentary	2	1	0	0
Crystalline	0	3	0	0
Volcanic	0	0	0	0
Mixed	0	0	0	0
Coastal Plain				
Sedimentary	1	0	5	0
Crystalline	0	0	0	0
Volcanic	0	0	0	0
Mixed	0	0	0	0

Note(s): Explanation of type is provided in Table 1.

## Discussion

If the USGS PA map (USGS 2003) were used to categorize wells or streams that are located in areas outside of PAs, then they would all be grouped into a single category: “other rocks.” In this paper, these areas were subdivided into 69 SHRs, thus providing a finer scale of resolution for assessing groundwater systems than was previously possible. In the discussion that follows, the locations of community supply wells in the conterminous U.S. are used to illustrate the utility of characterizing SHRs by type. The discussion also includes a summary of SHRs by lithology and geologic province, and presents some potential refinements and extensions that could be undertaken.

### Characterization of Wells in the Context of SHR Type

**Figure 5:**
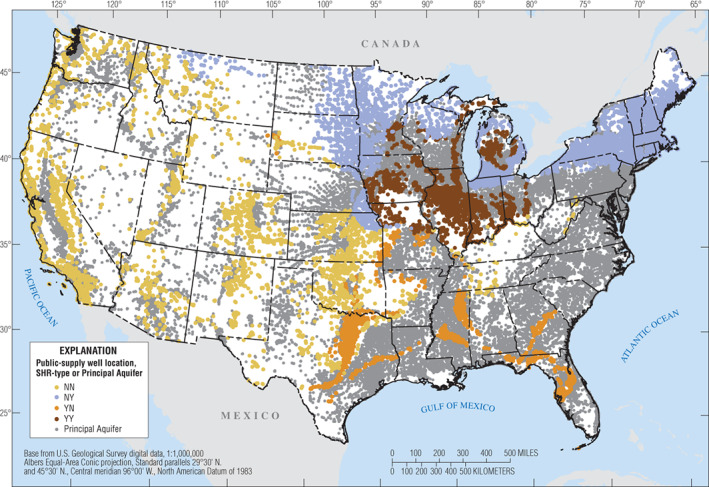
Map showing Community Supply Wells (Price and Maupin 2014) categorized by the type of Secondary Hydrogeologic Region (SHR) that the well plots within. Type indicates the relationship between an SHR and the presence or absence of underlying Principal Aquifers or overlying glacial deposits (see Table 1). There are about 143,000 wells shown: 9% are in SHRs classified as Type NN, 13% in Type NY, 3% in type YN, and 4% in Type YY; 71% are in Principal Aquifers.

Water wells are one of the primary means by which groundwater resources can be characterized. And given the three dimensional nature of groundwater systems, it is important to know an SHR's type (Table 1) because a well can extract water from the SHR, an underlying PA if present, the overlying glacial deposits if present, or some combination of the SHR, PA, and glacial deposits. If a well's two‐dimensional position, as indicated by its latitude and longitude, plots within the boundaries of an SHR classified as Type NN (Table 1), then it is likely that the well is tapping rocks within the SHR; wells plotting near boundaries may require additional inspection. SHRs classified as Type NN account for about 23% of the total area of the conterminous U.S. (Table 2), and about 9% of the community supply wells are located in these areas (Figure 5). Information from these community supply wells, such as water quality data, could be used to characterize groundwater resources in SHRs classified as Type NN. In this paper, these areas have been subdivided into 44 SHRs (Table 3).

If a well's two‐dimensional position plots within the boundaries of an SHR that is underlain by a PA and (or) overlain by glacial deposits, then the well might or might not tap rocks within the SHR. Additional inspection of the vertical position of the well screen or open interval would be needed. Comparable difficulties can also arise when categorizing wells that plot within the boundaries of a PA because PAs can also be overlain by glacial deposits and (or) underlain by other PAs. In the absence of information about which geologic units are tapped by a well, one could still categorize the well based upon its plotting position. In this paper, there are 26 SHRs identified as Type NY, YN, or YY, and they account for about 18% of the total area (Table 2) and about 20% of the community supply wells in the conterminous U.S. (Figure 5).

### SHRs Summarized by Lithology and Geologic Province

The lithology of an SHR is important because it provides an indication of the nature of the porosity and permeability of the rocks that comprise it, and because it can affect water quality. This is particularly important for SHRs that are Type NN; in these areas, one can generally expect wells to tap the volume of rock identified as an SHR. By definition, SHRs are not PAs, and are generally comprised of low permeability rocks or deposits.

Forty‐two of the 69 SHRs are comprised of sedimentary rocks (Table 3, Figure 4B), and these SHRs might include sandstones of limited spatial extent, or they might include carbonate rocks. Carbonates can be highly porous and permeable, particularly if the rocks have been exposed to secondary dissolution. In other cases, however, the porosity and permeability of carbonates can be quite low (Belitz and Bredehoeft 1988). As noted by Miller (2000), sedimentary rocks in areas mapped as other rocks can include shale, siltstone, evaporate deposits, silt, and clay. In some areas, sedimentary rocks can be fractured.

Fourteen of the 69 SHRS are comprised of crystalline rocks (Table 3, Figure 4B). If a well taps crystalline rocks, then the groundwater is generally expected to be transmitted through fractures; in many cases, storage occurs in the overlying sediments. Eight of the 14 crystalline SHRs are of Type NN, with seven located in the Cordilleran geologic province. The remaining six crystalline SHRs are Type NY, and are located either in the glaciated Central Interior subprovince or in the Appalachian part of the Appalachian‐Ouachita geologic province.

The remaining 13 SHRS are comprised of volcanic rocks or are classified as mixed, in which case there is no predominant lithologic class (Table 3, Figure 4B). All of the volcanic and mixed SHRs are Type NN, and 11 of the 13 are located in the Cordilleran geologic province. Porosity and permeability within these SHRs can be quite variable, and minor aquifers might or might not be present. SHRs comprised of mixed lithology could be subdivided on the basis of lithology.

Geologic provinces provide a context for assessing and understanding groundwater in areas mapped as SHRs (Table 3). In the Central Interior, three‐quarters of the SHRs are sedimentary and all four Types are represented. In the Cordilleran Mountain System, all of the SHRs are classified as Type NN and all four lithologic classes are represented. In the Appalachian‐Ouachita Mountain System, none of the SHRs are underlain by a PA and only two of the four lithologic classes are represented. In the Coastal Plain, all of the SHRs are sedimentary, and five of the six are underlain by a PA. The five SHRs classified as YN (Table 1) in the Coastal Plain can be characterized as confining layers and wells located in these SHRs are likely to extract water from an underlying PA.

### Possible Refinements and Extensions of the Current Work

The identification of SHRs is an important step in obtaining an improved understanding of the hydrogeologic framework of the conterminous U.S. Additional work can be pursued to develop a more refined understanding of that framework. Also, the newly developed framework can be used for purposes beyond characterization of groundwater accessed by wells.

The boundaries of the PAs as defined by the USGS (2003) are relatively generalized. The precision of the PA boundaries could be increased if one were to use the boundaries of geologic units as identified in the Geologic Map of North America (Garrity and Soller 2009). The criteria for identifying the PAs are defined in the Groundwater Atlas (Miller 2000), and those criteria could be used to identify the appropriate geologic units that correspond to the PAs. Alternatively, one could use the higher‐resolution State Geologic Map Compilation (Horton et al. 2017), but substantial effort would be required to resolve differences that may occur across state lines. The existing boundaries of PAs were respected in this paper in order to maintain consistency with previous work (USGS 2003).

The presence or absence of overlying glacial deposits was used to help classify SHRs (Table 1, Figure 4A). In this paper, the extent of glacial deposits was taken as the southernmost extent of Laurentide glaciation (Arnold et al. 2008). The depositional setting, thickness, and texture of deposits within the glaciated extent were not considered in the classification of the SHRs; these characteristics could be considered when evaluating groundwater resources in those SHRs overlain by glacial deposits. Identification of the vertical position of the well screen or open interval would be a critical part of such assessments.

Stream‐valley aquifers are not recognized as a PA (Miller 2000), nor do the SHRs account for the presence or absence of overlying stream‐valley aquifers. However, stream‐valley aquifers can be important sources of water supply (Sargent et al. 2008), particularly in the unglaciated Central Interior subprovince. Although stream‐valley aquifers have been generally mapped along major rivers and tributaries (Heath 1984; Miller 2000), they have not been mapped along smaller tributaries. In some cases, stream‐valley aquifers can extend across the boundaries of several PAs and (or) SHRs. The State Geologic Map Compilation (Horton et al. 2017) does provide a basis for a relatively comprehensive identification of stream‐valley aquifers, but it is beyond the scope of this paper to undertake that effort. In some assessments of PAs, stream‐valley aquifers were not separated from the larger PA. For example, assessment of groundwater quality in the High Plains PA included wells located within stream‐valley aquifers (Gurdak et al. 2009); in that study the stream‐valley aquifers were considered to be in hydrologic connection with the High Plains PA (Gutentag et al. 1984). Assessments of groundwater in SHRs that include overlying stream‐valley aquifers could implicitly incorporate those aquifers as has been done with PAs or additional work could be done to identify stream‐valley aquifers as appropriate.

The approach for identifying SHRs presented in this paper could be extended to areas of the U.S. that are outside of the conterminous states. Hawaii is underlain by a single PA (USGS 2003), and therefore no SHRs need be identified. Two PAs are identified in Puerto Rico, one in the Virgin Islands, and areas identified as other rocks in both territories are recognized as a minor aquifer (Miller et al. 1999); the minor aquifer could be defined as an SHR or it could be further subdivided. A single PA, consisting of unconsolidated deposits, is recognized in Alaska (USGS 2003). Areas of other rocks in Alaska include a wide range of lithologies, geologic ages, geologic and physiographic settings, and permafrost zones (Miller et al. 1999), and additional work would be required to identify SHRs.

The SHRs identified in this paper could be used to update previous work that identified Hydrologic Landscape Regions (HLRs) and assessed stream base flow in the context of HLRs (Wolock et al. 2004; Santhi et al. 2008). Those studies recognized six lithologic classes in the 60% of the conterminous U.S. that are mapped as PAs, and one lithologic class corresponding to other rocks which comprise the remaining 40%. Following the logic of the previous work and given the lithologies identified in the present work, four lithologic classes (Figure 4B)—crystalline, mixed, sedimentary, or volcanic—could be recognized in areas mapped as other rocks rather than just one. In addition, the previous work did not account for glacial deposits; future work could recognize glacial deposits and represent them using one or more lithologic classes. It is beyond the scope of this paper to re‐evaluate HLRs or to relate stream base flow to bedrock permeability.

## Summary

Classification of groundwater regions is important because it provides a foundation and context for a wide range of studies. The USGS has classified the Nation's groundwater, recognizing 57 Principal Aquifers in the conterminous U.S. (Miller 2000; USGS 2003). The USGS PA map identifies PAs only where they outcrop at the surface or subcrop beneath surficial deposits. Areas outside of the Principal Aquifers were simply identified as “not a principal aquifer” (Miller 2000) or as “other” (USGS 2003). For the purposes of discussion, areas outside of PAs are defined here as “other rocks.” It is important to subdivide areas of other rocks into smaller regions for several reasons: they account for 40% of the conterminous U.S.; 29% of the nation's community supply wells are located in these areas; and, stream base flow is not limited to areas mapped as PAs.

In this paper, areas mapped as other rocks were subdivided into 69 SHRs (Figure 3). SHRs typically consist of low permeability rocks or deposits, but can include locally productive aquifers. The basic building block for identifying SHRs were geologic units as identified on the Geologic Map of North America (Garrity and Soller 2009), with supplementary information obtained from the State Geologic Map Compilation (Horton et al. 2017), to insure that SHRs consist of rocks of comparable age and lithology. SHRs also were assembled using additional analog and digital maps to insure that the geologic units within an SHR are of comparable geologic or physiographic setting (Fenneman and Johnson 1946; Frezon and Finn 1988; Reed and Bush 2007; Coleman and Cahan 2012). In addition, SHRs were assembled so that they have a consistent relationship with respect to the presence or absence of underlying PAs and (or) overlying glacial deposits.

For the purposes of discussion and context, each SHR was classified using three criteria: (1) presence or absence of underlying PAs and (or) overlying glacial deposits, (2) primary lithology, and (3) geologic province and subprovince. The relationship between an SHR and underlying PAs or overlying glacial deposits is important because it provides some indication of whether a supply well is extracting water from rocks within the SHR. The lithology of an SHR provides an indication of the nature of the porosity and permeability of the rocks that comprise it, and can affect the quality of groundwater within it. Classification by geologic province and subprovince provides context for assessment and understanding.

The newly developed SHR map is a complement to the PA map previously published by the USGS (USGS 2003). SHRs are identified in areas where the PAs were not, and are not identified where the PAs were. The two maps, taken together, provide a national‐scale hydrogeologic framework for conducting a wide range of groundwater studies and research. Additional work can be done to refine and extend that framework.

## Authors' Note

The author(s) does not have any conflicts of interest to report.

## Appendix

Table A1 provides characteristics of each of the 69 Secondary Hydrogeologic Regions (SHRs): area, lithologic class, and type. A lower‐case “x” in the first position of an SHR name indicates an SHR that consists of rocks that are comparable or identical to an adjacent principal aquifer (PA); the text following the “x” is the name of the associated PA (Miller 2000). The SHRs with a lowercase “x” could be considered to be a part of the adjacent PA. Several of the SHRs include a suffix; Table A2 provides a description of the abbreviations used in the suffix.

**Table A1:** Characteristics of Secondary Hydrogeologic Regions (SHRs)

	SHR	Area	Lithologic Class	Type
Cordilleran, Western				
1	WA OR Coast Ranges—S	22,113	Sedimentary	NN
2	WA OR Coast Ranges—V	15,214	Volcanic	NN
3	xPugetSound	55	Sedimentary	NN
4	Northern Cascade Mountains	38,354	Crystalline	NN
5	Middle Cascade Mountains	14,060	Volcanic	NN
6	Middle & Southern Cascade Mtns	14,881	Volcanic	NN
7	Klamath Mountains	31,922	Crystalline	NN
8	Coast Ranges	64,749	Sedimentary	NN
9	Sierra Nevada	64,533	Crystalline	NN
10	Southern CA Mtns	24,997	Mixed	NN
Cordilleran, Intermountain				
11	Pacific NW Volcanics	46,165	Volcanic	NN
12	Blue Mountains	9182	Mixed	NN
13	Basin and Range Highlands	232,826	Mixed	NN
14	Grand Canyon	45,684	Sedimentary	NN
15	xColorado Plateau	2612	Sedimentary	NN
16	Rio Grande Highlands	30,269	Volcanic	NN
Cordilleran, Rocky Mountains				
17	Northern Rocky Mtns—X	134,612	Crystalline	NN
18	Northern Rocky Mtns—S	36,159	Sedimentary	NN
19	Northern Rocky Mtns—V	22,332	Volcanic	NN
20	Northern Rocky Mtns—Q	4489	Sedimentary	NN
21	Middle Rocky Mtns—X	19,373	Crystalline	NN
22	Middle Rocky Mtns—S	62,604	Sedimentary	NN
23	Middle Rocky Mtns—V	13,850	Volcanic	NN
24	Southern Rocky Mtns—X	45,402	Crystalline	NN
25	Southern Rocky Mtns—S	21,650	Sedimentary	NN
26	Southern Rocky Mtns—V	19,815	Volcanic	NN
Central Interior, unglaciated				
27	Black Hills‐X	2061	Crystalline	NN
28	Black Hills‐S	2583	Sedimentary	YN
29	Front Range	4337	Sedimentary	NN
30	Upper Cretaceous, unglaciated	189,739	Sedimentary	NN
31	Raton Basin	60,664	Sedimentary	NN
32	Eastern New Mexico	85,391	Sedimentary	NN
33	Interior Permian	168,054	Sedimentary	NN
34	Interior Pennsylvanian	68,586	Sedimentary	NN
35	OK Permian‐Pennsylvanian	3939	Sedimentary	YN
36	Ozarks Paleozoic	470,74	Sedimentary	YN
37	Ozarks‐X	693	Crystalline	NN
38	Arches‐unglaciated	69,242	Sedimentary	NN
39	Texas‐Triassic	12,177	Sedimentary	NN
40	Texas‐Cretaceous	2586	Sedimentary	NN
41	Texas‐Pennsylvanian	15,235	Sedimentary	NN
42	West TX—S	19,503	Sedimentary	NN
43	West TX—I	11,640	Volcanic	NN
44	Llano Uplift	8086	Mixed	NN
45	Edwards‐Trinity KT	65,046	Sedimentary	YN
Central Interior, glaciated				
46	Upper Cretaceous, glaciated	280,284	Sedimentary	NY
47	Canadian Shield	201,089	Crystalline	NY
48	North Central Interior—X	5535	Crystalline	NY
49	North Central Interior—PZ	3439	Sedimentary	NY
50	Arches‐glaciated	89,515	Sedimentary	YY
51	Outer Michigan Basin	56,088	Sedimentary	NY
52	Inner Michigan Basin	23,428	Sedimentary	YY
53	Adirondack Mountains	25,801	Crystalline	NY
54	Northern Appalachian Basin—PZ	84,421	Sedimentary	NY
55	NE‐KS Permian‐Pennsylvanian	31,510	Sedimentary	NY
56	IA‐MO Pennsylvanian	87,656	Sedimentary	YY
57	Illinois Basin	112,295	Sedimentary	YY
Appalachian ‐Ouachita				
58	Champlain‐Hudson Valleys—X	1441	Crystalline	NY
59	Champlain‐Hudson Valleys—X	15,078	Sedimentary	NY
60	Northern Appalachian Mtns—X	155,884	Crystalline	NY
61	Cape Cod and Islands	1894	Crystalline	NY
62	xValley&Ridge	5050	Sedimentary	NN
63	Ouachita Mountains	28,124	Sedimentary	NN
Coastal Plain				
64	Texas‐KT	21,100	Sedimentary	NN
65	MS Embayment—KT	17,576	Sedimentary	YN
66	Gulf Coastal‐T	27,377	Sedimentary	YN
67	Southeast Coastal Plain—T	15,260	Sedimentary	YN
68	Florida‐T	19,127	Sedimentary	YN
69	Florida‐Q	483	Sedimentary	YN

The number in the first column corresponds to the numbers on the Map of SHRs (Figure 3). Area is reported in square kilometers. Type refers to the presence of a Principal Aquifer beneath (first letter) or glacial deposits above an SHR (second letter); Y, yes, present, and N, no, not present. Mtns, Mountains.

**Table A2:** Abbreviations Used in Suffixes in Table A1

Abbreviation	Description
K	Cretaceous
PZ	Paleozoic
Q	Quaternary
S	Sedimentary
T	Tertiary
V	Volcanic
X	Crystalline
